# Phenotypic severity of homozygous *GCK* mutations causing neonatal or childhood-onset diabetes is primarily mediated through effects on protein stability

**DOI:** 10.1093/hmg/ddu360

**Published:** 2014-07-11

**Authors:** Anne Raimondo, Ali J. Chakera, Soren K. Thomsen, Kevin Colclough, Amy Barrett, Elisa De Franco, Alisson Chatelas, Huseyin Demirbilek, Teoman Akcay, Hussein Alawneh, The International NDM Consortium, Sarah E. Flanagan, Martijn Van De Bunt, Andrew T. Hattersley, Anna L. Gloyn, Sian Ellard, Mohammad A. Abduljabbar, Mahmoud Al-Zyoud, Syed Aman, Louise Bath, Parijat De, Neeta Deshpande, Erdem Durmaz, Frank Eickmeier, Nancy Samir Elbarbary, Marc Fillion, Sujatha M. Jagadeesh, Melanie Kershaw, Waqas I. Khan, Wojciech Mlynarski, Kathryn Noyes, Catherine J. Peters, Nick Shaw, Irina Tiron, Doga Turkkahraman, Lesley Turner, Khadiga Y. Eltonbary, Bilgin Yuksel

**Affiliations:** 1 Oxford Centre for Diabetes Endocrinology & Metabolism, University of Oxford, Oxford OX3 7LE, UK,; 2 Institute of Biomedical and Clinical Science, University of Exeter Medical School, Exeter EX2 5DW, UK,; 3 Macleod Diabetes and Endocrine Centre and,; 4 Molecular Genetics Laboratory, Royal Devon and Exeter NHS Foundation Trust, Exeter EX2 5DW, UK,; 5 Department of Paediatric Endocrinology, Diyarbakir Children State Hospital, Diyarbakir 21100, Turkey,; 6 Division of Pediatric Endocrinology, Dr Sadi Konuk Education and Research Hospital, Bakirkoy, Istanbul 34147, Turkey,; 7 Pediatric Endocrine Division, Queen Rania Al Abdullah Hospital for Children, King Hussein Medical Center, Royal Medical Services, Amman 11814, Jordan and; 8 Oxford NIHR Biomedical Research Centre, Churchill Hospital, Oxford OX3 7LE, UK

## Abstract

Mutations in glucokinase (*GCK*) cause a spectrum of glycemic disorders. Heterozygous loss-of-function mutations cause mild fasting hyperglycemia irrespective of mutation severity due to compensation from the unaffected allele. Conversely, homozygous loss-of-function mutations cause permanent neonatal diabetes requiring lifelong insulin treatment. This study aimed to determine the relationship between *in vitro* mutation severity and clinical phenotype in a large international case series of patients with homozygous *GCK* mutations. Clinical characteristics for 30 patients with diabetes due to homozygous *GCK* mutations (19 unique mutations, including 16 missense) were compiled and assigned a clinical severity grade (CSG) based on birth weight and age at diagnosis. The majority (28 of 30) of subjects were diagnosed before 9 months, with the remaining two at 9 and 15 years. These are the first two cases of a homozygous *GCK* mutation diagnosed outside infancy. Recombinant mutant GCK proteins were analyzed for kinetic and thermostability characteristics and assigned a relative activity index (RAI) or relative stability index (RSI) value. Six of 16 missense mutations exhibited severe kinetic defects (RAI ≤ 0.01). There was no correlation between CSG and RAI (*r*^2^ = 0.05, *P* = 0.39), indicating that kinetics alone did not explain the phenotype. Eighty percent of the remaining mutations showed reduced thermostability, the exceptions being the two later-onset mutations which exhibited increased thermostability. Comparison of CSG with RSI detected a highly significant correlation (*r*^2^ = 0.74, *P* = 0.002). We report the largest case series of homozygous *GCK* mutations to date and demonstrate that they can cause childhood-onset diabetes, with protein instability being the major determinant of mutation severity.

## INTRODUCTION

Homozygous mutations in the gene encoding the enzyme glucokinase (*GCK*) cause a rare form of permanent neonatal diabetes (PNDM; OMIM entry #606176) that requires lifelong insulin treatment. Only 12 cases have been reported to date and all were diagnosed within the first 9 months of life (1–7). Glucokinase (GCK) acts as the pancreatic glucose sensor, and biallelic inactivation severely compromises the ability of the pancreatic β-cell to regulate insulin secretion in response to a glucose challenge. Treatment with sulphonylureas has been shown to augment insulin production in a single case report (4), but there are no reports of patients with homozygous *GCK* mutations who do not require insulin treatment. In contrast, heterozygous inactivating *GCK* mutations are less deleterious and manifest in a mild fasting hyperglycemia from birth (5.5–8 mmol l^−1^) otherwise known as maturity-onset diabetes of the young; subtype GCK (GCK-MODY; OMIM entry #125851) (8). Pharmacological treatment for these individuals is not usually required (9).

Functional characterization has shown a broad range of *in vitro* defects for >70 naturally occurring GCK-MODY mutations, with a particular emphasis on kinetic effects (8). Protein instability has also been shown to contribute to enzyme dysfunction in some cases via effects on enzyme turnover (10–20). Individuals with heterozygous *GCK* mutations have a remarkably consistent phenotype due to compensation by the wild-type (WT) allele, which is posttranslationally upregulated by glucose (21). This means that the true relationship between *in vitro* mutation severity and clinical phenotype can only be investigated in patients with homozygous or compound heterozygous *GCK* mutations.

In this study, we aimed to establish the molecular mechanisms driving *GCK* dysfunction through the evaluation of a large international case series of patients with homozygous *GCK* mutations. We identified 19 unique mutations (16 missense, 2 frameshift, 1 deletion) in 30 patients: 28 patients with PNDM and 2 with childhood-onset diabetes (diagnosed at age 9 and 15 years). These latter two individuals were referred for genetic testing for MODY and are the first two reported patients with a homozygous *GCK* mutation diagnosed outside infancy. Combined with extensive clinical data, we were able to correlate functional impact with clinical phenotype for the 16 missense mutations by analyzing their effects on enzymatic activity and thermostability *in vitro*. We discovered that protein instability was more highly correlated with phenotypic severity than kinetic dysfunction, providing the first corroborative evidence that enzyme turnover may be a vital contributor to physiological *GCK* mutational effects.

## RESULTS

### The cohort

We studied a cohort of 30 patients with 19 unique homozygous GCK mutations (Table 1) (22). The mutations identified were typically found in communities with high rates of consanguinity: patients were mostly of Arabic, Turkish, Indian or Pakistani ancestry. Twenty-eight of these patients have PNDM, and two were diagnosed with diabetes aged 9 or 15 years, consistent with MODY. All five individuals with the p.R397L mutation are British Pakistanis, and the two individuals with childhood-onset diabetes (i.e. the homozygous p.D160N and p.V226M carriers) are white Canadians. Interestingly, in our cohort of GCK-MODY individuals, over a quarter (12 of 46) of Canadian probands have a heterozygous p.V226M GCK mutation, all of whom are French Canadian (9). There is only one other proband in our cohort (comprising >1200 individuals) with a heterozygous p.V226M GCK mutation. Six of the 16 homozygous missense mutations are novel (c.148C>G, p.H50D; c.491T>C, p.L146P; c.451T>A, p.S151T; c.506A>G, p.K169R; c.1178T>C, p.M393T; and c.1322C>T, p.S441L), as are the two duplication mutations (c.764_767dup, p.E256fs and c.1121dup, p.S375fs) and one deletion mutation (c.1256del, p.F419fs). The duplication/deletion mutations result in a premature termination codon predicted to reduce protein levels and were not further investigated. Four patients have previously been described [both patients with a p.T168A mutation (4,7), one patient with a p.R397L mutation (3) and the patient with a p.G261R mutation (6)].

**Table 1.** Clinical characteristics of patients with a homozygous *GCK* mutation

Mutation	Cases	BW (g)	Gestational age (weeks)	BW SDS	Age at diagnosis (days)	Glucose at diagnosis (mmol l^−1^)	C peptide (ng ml^−1^)	HbA1c (mmol mol^−1^ [%])	Insulin dose (units kg^−1^ day^−1^)	Country
p.E40K (c.118G>A)	1	1500	38	−3.93	112	25	<0.1	123 [13.4]	1.3	Pakistan
p.R43C (c.127C>T)	1	2300	37	−1.50	161	23.1	N/A	79 [9.4]	1	India
p.H50D (c.148C>G)	2	1425 (1250–1600)	39 (38–39)	−4.63 (−4.41–−4.85)	16 (3–28)	31 (23–39)	0.5 (0.1–0.9)	72 (54–89) [8.7 (7.1–10.3)]	1.1^a^	Egypt
p.G72R (c.214G>A)	1	2240	38	−2.13	1	7.9	0.13	N/A	0.3	Turkey
p.L146P (c.491T>C)	1	1465	35	−2.53	1	24	0.11	69 [8.5]	0.8	Qatar
p.S151T (c.451T>A)	1	1700	40	−4.71	42	N/A	N/A	64 [8.0]	1	India
p.D160N (c.478G>A)	1	3285	N/A^c^	−0.16	3287	6.0	N/A	65 [8.1]	None	Canada
p.T168A (c.502A>G)	2	1400^a^	37^a^	−3.67^a^	151 (56–245)	17.3 (16.5–18.0)	N/A	82 (63–100) [9.6 (7.9–11.3)]	1 (0.8–1.1)	Turkey
p.K169R (c.506A>G)	3	1750 (1600–1900)	39 (38–40)	−3.82 (−3.67–−3.94)	11 (1–28)	39.8 (28.0–48.0)	0.29 (0.08–0.5)	63 (62–63) [7.9 (7.8–7.9)]^a^	1.4 (1.3–1.5)^a^	Turkey
p.A208T (c.623C>T)	2	1675 (1450–1900)	38 (36–40)	−3.4 (−2.96–−3.85)	18 (0–35)	26.7 (13.8–39.6)	N/A	72 (69–74) [8.7 (8.5–8.9)]	1.2 (1.0–1.3)	Poland
p.V226M (c.676G>A)	1	3500	40	0.203	5479	N/A	N/A	N/A	None	Canada
p.E256fs (c.764_767dup)	1	1600	38	−3.75	21	N/A	N/A	N/A	N/A	India
p.G261R (c.781G>A)	1	2400	39	−2.22	7	20	N/A	89 [10.3]	1	Saudi Arabia
p.S375fs (c.1121dup)	1	1500	36	−2.92	140	20	<0.10	135 [14.5]	N/A	Pakistan
p.M393T (c.1178T>C)	1	2350	38	−1.87	21	22.5	0.38	N/A	1	Turkey
p.R397L (c.1190G>T)	5	1666 (1370–1820)	37 (36–38)	−3.08 (−2.1–−3.51)	24 (2–84)	21 (11–33)^a^	N/A	83 (64–100) [9.7 (8.0–11.3)]^a^	1.1 (1.0–1.2)^b^	UK/Pakistan
p.F419fs (c.1256del)	1	1400	36	−3.17	14	18	N/A	89 [10.3]	1.3	Turkey
p.S441L (c.1322C>T)	1	2500	38	−1.53	84	17	N/A	83 [9.7]	1.3	Turkey
p.A449T (c.1345G>A)	3	3233 (3000–3700)	41 (40–42)	−1.17 (−1.92–0.32)	98 (21–126)	17.4 (13.8–22)	0.30 (0.05–0.55)^a^	59 (54–64) [7.6 (7.1–8.0)]	0.9 (0.7–1.0)	Jordan

Mutations are described according to *Homo sapiens* *GCK* reference sequence NM_000162.3. Where there is data for more than one patient per mutation, the mean and range are shown. The indicated range in BW SDS refers to the range of birth weight for that mutation. BW, birth weight; SDS, standard deviation score; N/A, data not available.

^a^Data unavailable for one individual.

^b^Data unavailable for two individuals.

^c^Presumed ‘at term’ for calculation of BW SDS.

**Figure 1.**
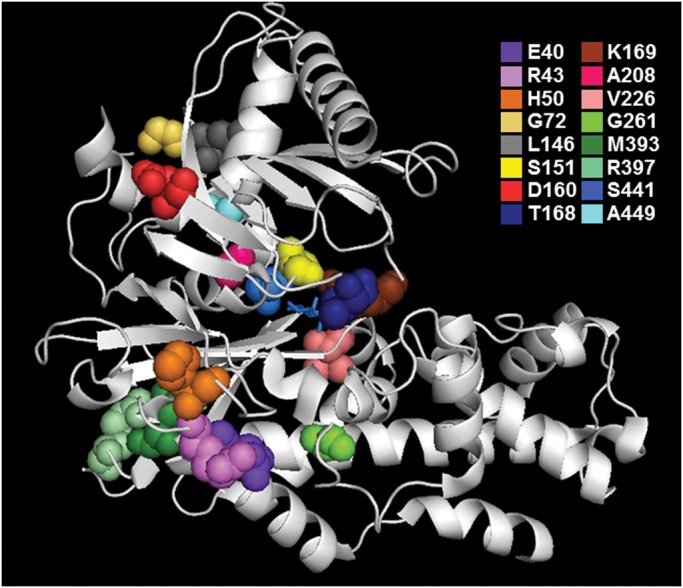
Ribbon model of the closed (glucose-bound) form of human GCK illustrating each of the 16 missense mutations. Glucose is indicated in stick form in the center of the active site.

Preliminary *in silico* analyses using the PolyPhen-2, SIFT and Condel algorithms predicted all 16 missense mutations to be damaging, with the exception of p.H50D and p.D160N, which produced conflicting predictions (Supplementary Material, Table S1) (23–25). We also mapped each missense mutation onto the crystal structure of β-cell GCK and found that several mutations were located within or proximal to the glucose and/or adenosine triphosphate (ATP) binding sites (Fig. 1) (26).

### Clinical features of neonatal diabetes patients

The majority (22 of 28) of patients with neonatal diabetes were diagnosed within the first 3 months of life, with 10 diagnosed within the first week. The median age of diagnosis was 21 days (range 0–245 days). The median birth weight was 1700 g (range 1250–3700 g) with 18 of the 28 patients having a birth weight SDS below −2.0 (equivalent to the 1st centile). Three of 13 patients tested for fasting C-peptide showed signs of preserved β-cell function, as defined by a cut-off score of ≥0.23 ng ml^−1^ derived from the Diabetes Control and Complications Trial (27). However, all patients required insulin treatment, with the median dose being 1 unit kg^−1^ day^−1^ (range 0.7–1.3 units). One previously reported patient was also treated with glibenclamide (4). Median HbA1c established on treatment for all 22 patients was 72 mmol mol^−1^ [8.7%] (range 54–135 mmol mol^−1^ [7.1–14.5%]) (Table 1).

### Clinical features of childhood-onset diabetes patients

The first patient was diagnosed with diabetes at 9 years of age. She had asymptomatic, high fasting blood glucose readings for 3 years with mildly elevated postprandial glucose and was not on pharmacological treatment. DNA was provided to investigate the likelihood of a heterozygous *GCK* mutation but unexpectedly revealed the presence of a homozygous mutation, c.478G>A, p.D160N. This result was confirmed by Sanger sequencing using alternative primers to check for allelic drop out. Analysis of parental DNA confirmed that both parents were heterozygous for the same mutation. Their fasting glucose levels were also mildly elevated, consistent with a diagnosis of GCK-MODY (Supplementary Material, Fig. S1A).

The second patient was diagnosed with diabetes at 15 years of age. She was treated with a basal-bolus insulin regime on the basis of a presumed diagnosis of type 1 diabetes. However, given a lack of GAD65/IA2 antibodies and a maternal family history of diabetes for three generations (Supplementary Material, Fig. S1B), she was referred for *HNF1A* and *HNF4A* genetic testing. No mutation was found but further testing on a targeted next-generation sequencing platform (28) revealed a homozygous *GCK* mutation, c.676G>A, p.V226M.

### Developing a clinical severity scoring system

To determine which clinical features could be used to define severity of disease for this case series, we analyzed the linear correlation for several clinical markers (HbA1c, insulin dose day^−1^kg^−1^, age at diagnosis, fasting C-peptide) against birth weight standard deviation score (BW SDS) for all individuals. We chose BW SDS as the reference variable for clinical severity as it reflects insulin-mediated growth, which is dependent on fetal insulin secretion *in utero* and is therefore a reliable, independent indicator of *GCK* mutational severity. Only age at diagnosis showed a significant linear correlation with BW SDS (*r*^2^ = 0.33, *P* = 0.001) (Supplementary Material, Fig. S2). The other factors either had insufficient clinical data for robust statistical analysis (C-peptide, data not shown) or were haphazardly distributed [HbA1c (*r*^2^ = 0.01, *P* = 0.6), insulin dose day^−1^kg^−1^ (*r*^2^ = 0.16, *P* = 0.07)], perhaps indicative of variable concordance with treatment or insufficient contact between patients and their referring clinicians. We therefore assigned a clinical severity score (CSS) to each patient based on degrees of BW SDS and age at diagnosis, and used this information to allocate each mutation to one of four graded categories according to their cumulative score (Supplementary Material, Table S2).

### Developing a functional severity scoring system

**Figure 2.**
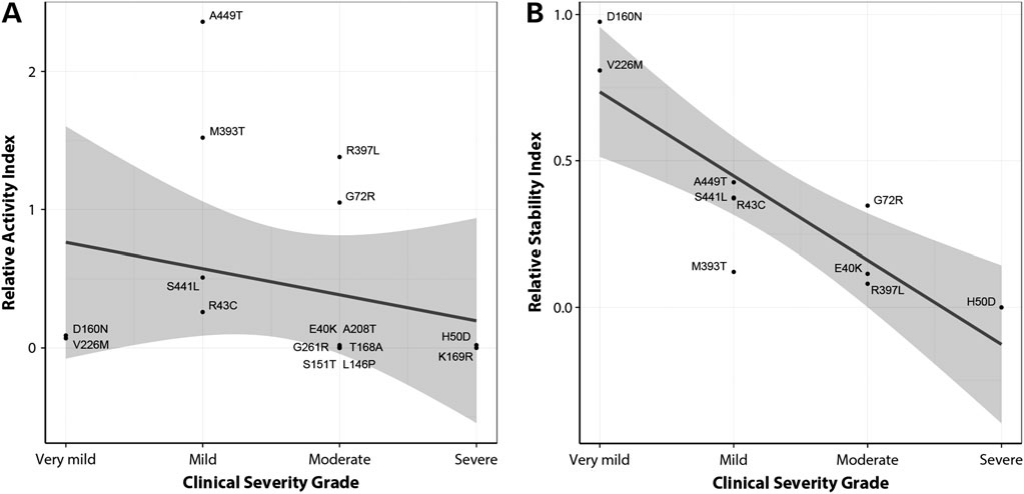
Linear regression analysis of Clinical Severity Grade against (**A**) RAI or (**B**) RSI for GCK missense mutations. The 95% confidence intervals for the linear regression lines are shaded in gray.

To establish whether there was any link between clinical severity and *in vitro* enzyme characteristics, we performed functional analysis on all 16 missense mutations using the previously characterized neutral rare variants p.G68D and p.T342P as controls for WT-like activity (29,30). Fourteen missense mutations displayed inactivating kinetics, including four mutations that retained <10% activity (relative activity index, RAI < 0.1) relative to WT-GCK, and six mutations that were so kinetically deficient that they retained 1% activity or less (RAI ≤ 0.01) (Supplementary Material, Table S3). The remaining mutations displayed minimal loss of activity, and in the case of p.A449T was paradoxically mildly kinetically activating. The two childhood-onset mutations (p.D160N and p.V226M) were among those that retained <10% WT activity. There was no linear correlation between RAI and clinical severity grade (CSG) (*r*^2^ = 0.05, *P* = 0.39), demonstrating that kinetic characteristics alone were insufficient to explain the PNDM phenotype (Fig. 2A).

**Figure 3.**
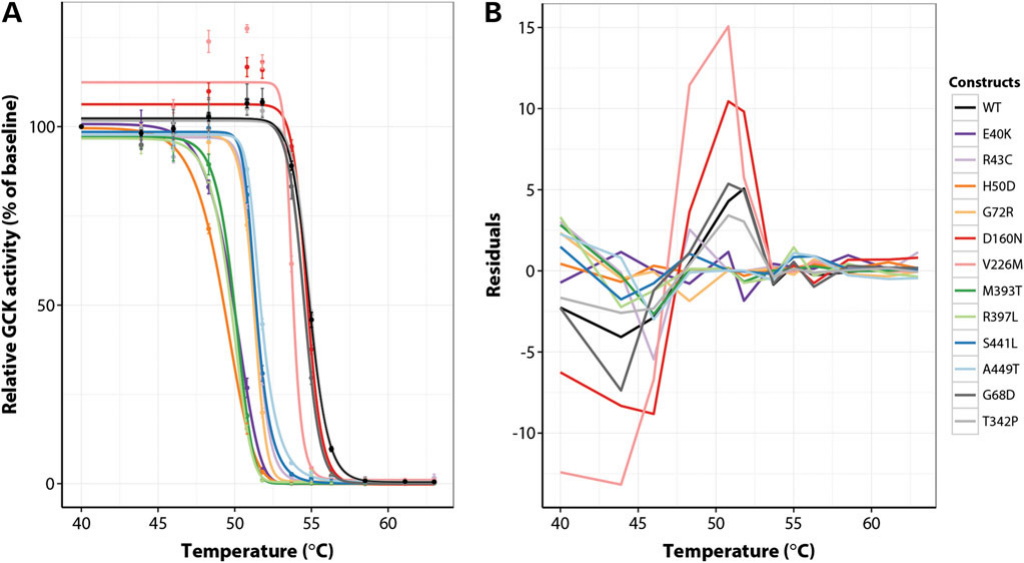
Assessment of thermostability for WT and mutant human GST-GCK proteins. Logistic regression modelling was used to fit an activity curve to each protein in (**A**). Data were normalized to the baseline level of activity for each protein at 40°C. Each point represents mean activity ± SEM (*n* = 3 experiments except for WT and p.T342P *n* = 10 and p.D160N *n* = 6). The raw residuals, defined as the difference between observed and predicted values, for all proteins are shown in (**B**).

Given the lack of correlation with kinetic characteristics, we explored other mechanisms of enzyme dysfunction, and investigated the behavior of every mutant GCK protein that displayed >1% activity relative to WT-GCK (RAI > 0.01; a total of 10 mutants) in thermostability assays. Thermal instability has been shown to be indicative of reduced cellular GCK protein expression (11,20). Across a temperature range of 40–63°C, WT, p.G68D and p.T342P-GCK retained at least 100% activity up to 51.8°C, after which their activity dropped dramatically due to thermal denaturation (Fig. 3A). Eight of 10 mutants displayed markedly inferior thermostability characteristics, as indicated by loss of activity at much lower temperatures (Fig. 3A). The behavior of these eight proteins could be accurately captured by logistic regression models, and examination of the residual plot for each protein confirmed the appropriateness of this approach, as we observed an excellent match (i.e. small, randomly distributed differences only) between the observed activities at each temperature point and those predicted by the fitted models (Fig. 3B). For WT, p.G68D and p.T342P-GCK, however, systematic differences were seen between the observed and predicted activities, mainly due to an increase in activity for these proteins up to 51.8°C (Fig. 3A and B). This increase was also seen for the two childhood-onset mutations (p.D160N and p.V226M) but to a much greater extent, resulting in significantly higher activities for these two mutants at 51.8°C compared with WT (*P* < 0.001 for both proteins, Student's *t*-test). These results suggest that the p.D160N and p.V226M substitutions confer an atypical stability profile to the GCK protein that may be indicative of enhanced cellular stability *in vivo*.

We assigned each mutant a relative stability index (RSI) value, which was calculated from the temperature point at which each mutant protein displayed 50% activity (TA50) (Supplementary Material, Table S3). Even though this approach did not take into account the greater activity maxima for the p.D160N and p.V226M proteins compared with WT, there was a highly significant linear correlation between CSG and RSI (*r*^2^ = 0.74, *P* = 0.002) (Fig. 2B), indicating that increased clinical (and hence mutational) severity is related to the underlying degree of protein instability in this dataset.

## DISCUSSION

Here, we report a series of 30 patients with 19 unique homozygous *GCK* mutations, including the first two cases of a homozygous mutation diagnosed with diabetes outside infancy (aged 9 and 15 years). This study significantly extends the phenotypic spectrum of naturally occurring homozygous *GCK* mutations, and utilizes both clinical and *in vitro* approaches to provide the first systematic investigation of genotype–phenotype correlations within a large group of patients with homozygous *GCK* mutations. We demonstrate that these mutations commonly affect *GCK* by altering enzyme stability as well as kinetics, and that a significant correlation with phenotypic severity was only revealed when both were considered. This was particularly the case for the childhood-onset p.D160N and p.V226M mutations, which displayed inactivating kinetics indistinguishable from the neonatal-onset mutations, but thermostability characteristics indicative of ‘super-stable’ proteins, thereby suggesting that improved protein stability may ameliorate disease severity by increasing the available pool of GCK protein. Further studies will be needed to characterize the cellular phenotype of these proteins more fully.

Our study brings the total number of homozygous *GCK* cases described worldwide to 38 (1–7). The clinical phenotype of the GCK-PNDM individuals in our case series is similar to that observed in the literature to date: very low birth weight, typically <2.5th centile; diagnosis of diabetes within the first few months of life; and insulin treatment required. The childhood-onset c.478G>A, p.D160N mutation has been reported in the heterozygous state in six other cases of GCK-MODY and co-segregated with raised fasting glucose in these families (8). Similarly, the c.676G>A, p.V226M mutation has also been previously reported in 12 families where a heterozygous mutation co-segregated with raised fasting glucose in a manner consistent with GCK-MODY in at least two generations (8).

The unexpected functional results of this study suggest that protein stability should be more rigorously explored as a key mechanism of GCK inactivation. This has been previously proposed for some naturally occurring GCK-MODY mutations (10–19,21,31), including a few that have also been studied in mice (20,32), but has never been systematically investigated in such a large group of patients with homozygous *GCK* mutations. Furthermore, our results indicate that protein stability may be the principal determinant of phenotypic severity for all but the most severely kinetically defective mutations. We identified six mutations (p.L146P, p.S151T, p.T168A, p.K169R, p.A208T and p.G261R) with negligible activity in kinetic assays, four of which map directly to the glucose binding site of GCK (Fig. 1; Supplementary Material, Table S3). It is reasonable to predict that these mutations would be essentially unresponsive to glucose in a cellular context and could therefore be considered ‘null’ mutations solely on the basis of their kinetic characteristics, although it is possible that they may also be thermolabile (11). Individuals with these mutations possessed among the highest CSSs, suggesting that they may indeed retain minimal GCK functionality (Supplementary Material, Table S2). Our statistical analyses, however, indicated that the overall severity of the remaining mutations was more readily explained by their thermostability characteristics, suggesting that the cell may be relatively tolerant to loss of GCK function provided that a sufficient ‘steady state’ pool of readily accessible enzyme is maintained. Studies in homozygous or compound heterozygous *Gck* mutant mice have also found a correlation between severity of hyperglycemia and protein stability via thermal shift experiments, pointing towards a key role for enzyme turnover in determining disease severity (20,32).

In summary, we present the largest case series of homozygous *GCK* mutations reported to date, and demonstrate for the first time that clinical presentation of diabetes is determined by *in vitro* mutation severity, with milder mutations causing childhood-onset diabetes. Homozygous *GCK* mutations are thus a rare cause of childhood-onset diabetes and could be considered in consanguineous or isolated populations. Furthermore, we demonstrate that the major determinant of mutation severity, except in cases where a mutation completely abolishes kinetic activity, is protein instability.

## MATERIALS AND METHODS

### Study subjects

We collated clinical details for 30 patients with diabetes due to homozygous *GCK* mutations. The two childhood-onset cases were identified through diagnostic genetic testing for MODY. Parent and family details are routinely collected as part of the neonatal diabetes service to facilitate an appropriate testing strategy. Where there were missing family details, we contacted the referring clinicians to request additional information.

### GCK screening and mutation identification

We screened the β-cell isoform of *GCK* (NM_000162.3) in 22 patients with neonatal diabetes by Sanger sequencing in consanguineous pedigrees. A further six cases of genetically undiagnosed neonatal diabetes were screened on the Illumina HiSeq2000 targeted next-generation sequencing platform covering all known monogenic diabetes genes (28). One of the two patients with childhood-onset diabetes was diagnosed by Sanger sequencing during routine genetic testing and the other by next-generation sequencing as described above. All identified sequence variants were submitted to the Leiden Oven Variant Database for *GCK* (www.lovd.nl/GCK, last accessed on 19 May 2014).

### Clinical and laboratory analyses

Patients' clinicians were contacted by email to identify clinical details including birth weight, gestational age, age at diabetes diagnosis, HbA1c, glucose at diagnosis, C-peptide with matched glucose, current treatment and insulin dose corrected for weight and ethnicity. Clinical details for each of the patients' mutations are given in Table 1.

A CSS for each patient was subsequently calculated based on degrees of BW SDS and age at diagnosis (Supplementary Material, Table S2). BW SDS, according to World Health Organization guidelines (http://www.rcpch.ac.uk/growthcharts, last accessed on 24 March 2014), was first divided into quartiles. Patients in the highest birth weight quartile scored 1, whereas those in the lowest quartile scored 4. Age at diagnosis was scored such that diagnosis within 1 week scored 4, within 1 month scored 3, within 6 months scored 2 and the remainder scored 1. The cumulative CSS for each patient was the sum of their BW SDS score and their age at diagnosis score, with a maximum possible total score of 8. Where more than one patient was identified with the same *GCK* mutation, individual CSSs were averaged by mutation to give a mutation-specific CSS.

A CSG was assigned to each mutation according to its CSS. The maximum possible total score was first divided into four grades. Those with CSS < 2 were designated ‘Very Mild’, those with 2 < CSS ≤ 4 were designated ‘Mild’, those with 4 < CSS ≤ 6 were designated ‘Moderate’ and those with CSS > 6 were designated ‘Severe’.

### Cloning and mutagenesis

Mutations were introduced into the β-cell *GCK* variant (17) via site-directed mutagenesis using the Stratagene QuikChange II kit (Agilent Biotechnologies) according to the manufacturer's instructions. All plasmid sequences were verified by sequencing. All primers were obtained from Eurofins Genetic Services Ltd Primer sequences are available upon request.

### Protein production

Production of GST-tagged WT and mutant GCK proteins has been described previously (17,33).

### Functional characterization

GCK activity was measured spectrophotometrically using glucose 6-phosphate dehydrogenase-coupled assays essentially as described (16,17). Affinity for glucose was measured in the presence of 0–100 mmol l^−1^ glucose, except for the particularly deleterious mutants p.E40K and p.H50D where it was increased to 0–200 mmol l^−1^; p.V226M 0–500 mmol l^−1^; and p.T168A, p.A208T and p.G261R 0–1000 mmol l^−1^. Affinity for ATP was determined in the presence of 0–5 mmol l^−1^ ATP, except for the particularly deleterious mutants p.G72R and p.A449T where it was increased to 0–10 mmol l^−1^; p.T168A and p.V226M 0–25 mmol l^−1^; and p.A208T 0–20 mmol l^−1^.

Thermostability assays were conducted essentially as described (16, 18). Each variant was analyzed over a 12-point temperature gradient spanning 40–63°C. All variants were analyzed at a final glucose concentration of 8 mmol l^−1^ except for the p.E40K and p.H50D variants, which were analyzed at 22 mmol l^−1^ glucose, and the p.V226M variant, which was analyzed at 45 mmol l^−1^ glucose. WT-GCK and the control variant p.T342P-GCK were analyzed at all glucose concentrations. The thermostability characteristics of these proteins did not alter in response to glucose concentration.

### Graphical and statistical analyses

Glucose affinity (S0.5), Hill number (nH) and turnover number (*K*_cat_) values were calculated using the Hill equation. ATP affinity (ATPKm) was calculated using the Michaelis–Menten equation. All data fits were performed using Kaleidagraph v3.52 (Synergy Software). Relative activity indices were calculated using the equation first described by Christesen *et al.*, which normalizes to a blood glucose of 5 mmol l^−1^ (*K*_cat_ values were taken from the glucose S0.5 assay) (34). Relative stability indices were defined as (TA50(mutant) − TA50(min))/(TA50(WT) − TA50(min)), where TA50 refers to the temperature point at which each protein displayed 50% of its activity at 40°C and TA50(min) refers to the minimum observed TA50 of any construct in this assay.

For comparison of clinical markers with BW SDS, linear correlation analyses were conducted using Stata 13.1. The relationship between CSG and RAI or RSI was analyzed via linear regression in R 3.0.2. All other clinical data analyses—including calculation of medians and quartiles of BW SDS—were performed using Stata 13.1. For thermostability assays, a five-parameter logistic regression model was used to fit thermostability data and calculate raw residuals and TA50 values for each mutant in R 3.0.2.

### Bioinformatic analyses

Predicted pathogenicity for all variants was assessed using the PolyPhen v.2.2.2, SIFT Human Protein, and Condel web server algorithms (23–25).

### Structural modelling

Variants were mapped onto the crystal structure of human GCK bound to glucose (closed form; Protein DataBank entry 1V4S) using PyMOL v.0.99.

## SUPPLEMENTARY MATERIAL

Supplementary Material is available at *HMG* online.

## FUNDING

This work was supported by the Wellcome Trust (grant number 095101/Z/10/Z to A.L.G.). A.L.G. is a Wellcome Trust Senior Fellow in Basic and Biomedical Science. A.T.H. and S.E. are Wellcome Trust Senior Investigators. A.T.H. is a National Institute for Health Research (NIHR) Senior Investigator. A.T.H. is funded by the NIHR Exeter Clinical Research Facility. This article presents independent research supported by the NIHR Exeter Clinical Research Facility. The views expressed are those of the authors and not necessarily those of the NHS, the NIHR or the Department of Health. Funding to pay the Open Access publication charges for this article was provided by the Wellcome Trust.

## Supplementary Material

Supplementary Data

## Appendix

The International Neonatal Diabetes Mellitus Consortium

Mohammad A. Abduljabbar^1^, Mahmoud Al-Zyoud^2^, Syed Aman^3^, Louise Bath^4^, Parijat De^5^, Neeta Deshpande^3^, Erdem Durmaz^6^, Frank Eickmeier^7^, Nancy Samir Elbarbary^8^, Marc Fillion^9^, Sujatha M. Jagadeesh^10^, Melanie Kershaw^11^, Waqas I. Khan^12^, Wojciech Mlynarski^13^, Kathryn Noyes^4^, Catherine J. Peters^14^, Nick Shaw^15^, Irina Tiron^16^, Doga Turkkahraman^6^, Lesley Turner^17^, Khadiga Y. Eltonbary^18^, Bilgin Yuksel^19^

1. Pediatric Endocrinology Services, Department of Pediatrics, Dhahran Health Center, Saudi Aramco, Dhahran 31311, Saudi Arabia
2. Division of Pediatric Endocrinology, Hamad Medical Corporation, PO Box 3050, Doha, Qatar
3. Belgaum Diabetes Centre, Maruti Galli, Belgaum 590001, Karnataka, India
4. Department of Diabetes, Royal Hospital for Sick Children, Edinburgh, EH9 1LF, UK
5. Diabetes and Endocrine Unit, City Hospital, Dudley Road, Birmingham B18 7QH, UK
6. Department of Pediatric Endocrinology, Akdeniz University Hospital, Antalya 07985, Turkey
7. Oberarzt Klinik für Kinder und Jugendliche, Klinikum Pforzheim GmBH, Pforzheim 75175, Germany
8. Diabetes and Endocrinology Unit, Department of Pediatrics, Faculty of Medicine, Ain shams University, Cairo 11361, Egypt
9. Département de Pédiatrie, CHUQ-CHUL-Centre-Mère-Enfant, Quebec G1V 4G2, Canada
10. Department of Clinical Genetics, MediScan Systems, Mylapore, Chennai 600004, India
11. George Eliot Hospital, College Street, Nuneaton CV10 7DJ, UK
12. Department of Pediatric Medicine, The Children's Hospital and The Institute of Child Health, Multan 60000, Pakistan
13. Department of Paediatrics, Oncology, Haematology and Diabetology, Medical University of Lodz, Sporna 36/50, 91-738 Lodz, Poland
14. Department of Endocrinology, Great Ormond Street Hospital for Children, London WC1N 3JH, UK
15. Department of Endocrinology and Diabetes, Birmingham Children's Hospital NHS Foundation Trust, Birmingham B4 6NH, UK
16. Heartlands Hospital, Heart of England NHS Foundation Trust, Bordesley Green East, Birmingham B9 5SS, UK
17. James Paton Memorial Hospital, Gander, Newfoundland, Canada A1V 1P7
18. Pediatric Department, Ain Shams University, Abassia, Cairo 1156, Egypt
19. Division of Pediatric Endocrinology and Metabolism, Çukurova University, Balcali, Adana 01330, Turkey

## References

[1] Njolstad P.R., Sovik O., Cuesta-Munoz A., Bjorkhaug L., Massa O., Barbetti F., Undlien D.E., Shiota C., Magnuson M.A., Molven A. (2001). Neonatal diabetes mellitus due to complete glucokinase deficiency. N. Engl. J. Med..

[2] Njolstad P.R., Sagen J.V., Bjorkhaug L., Odili S., Shehadeh N., Bakry D., Sarici S.U., Alpay F., Molnes J., Molven A. (2003). Permanent neonatal diabetes caused by glucokinase deficiency: inborn error of the glucose-insulin signaling pathway. Diabetes.

[3] Porter J.R., Shaw N.J., Barrett T.G., Hattersley A.T., Ellard S., Gloyn A.L. (2005). Permanent neonatal diabetes in an Asian infant. J. Pediatr..

[4] Turkkahraman D., Bircan I., Tribble N.D., Akcurin S., Ellard S., Gloyn A.L. (2008). Permanent neonatal diabetes mellitus caused by a novel homozygous (T168A) glucokinase (GCK) mutation: initial response to oral sulphonylurea therapy. J. Pediatr..

[5] Rubio-Cabezas O., Diaz Gonzalez F., Aragones A., Argente J., Campos-Barros A. (2008). Permanent neonatal diabetes caused by a homozygous nonsense mutation in the glucokinase gene. Pediatr. Diabetes.

[6] Bennett K., James C., Mutair A., Al-Shaikh H., Sinani A., Hussain K. (2011). Four novel cases of permanent neonatal diabetes mellitus caused by homozygous mutations in the glucokinase gene. Pediatr. Diabetes.

[7] Durmaz E., Flanagan S., Berdeli A., Semiz S., Akcurin S., Ellard S., Bircan I. (2012). Variability in the age at diagnosis of diabetes in two unrelated patients with a homozygous glucokinase gene mutation. J. Pediatr. Endocrinol. Metab..

[8] Osbak K.K., Colclough K., Saint-Martin C., Beer N.L., Bellanne-Chantelot C., Ellard S., Gloyn A.L. (2009). Update on mutations in glucokinase (GCK), which cause maturity-onset diabetes of the young, permanent neonatal diabetes, and hyperinsulinemic hypoglycemia. Hum. Mutat..

[9] Stride A., Shields B., Gill-Carey O., Chakera A.J., Colclough K., Ellard S., Hattersley A.T. (2013). Cross-sectional and longitudinal studies suggest pharmacological treatment used in patients with glucokinase mutations does not alter glycaemia. Diabetologia.

[10] Matschinsky F.M. (2002). Regulation of pancreatic beta-cell glucokinase: from basics to therapeutics. Diabetes.

[11] Burke C.V., Buettger C.W., Davis E.A., McClane S.J., Matschinsky F.M., Raper S.E. (1999). Cell-biological assessment of human glucokinase mutants causing maturity-onset diabetes of the young type 2 (MODY-2) or glucokinase-linked hyperinsulinaemia (GK-HI). Biochem. J..

[12] Galan M., Vincent O., Roncero I., Azriel S., Boix-Pallares P., Delgado-Alvarez E., Diaz-Cadorniga F., Blazquez E., Navas M.A. (2006). Effects of novel maturity-onset diabetes of the young (MODY)-associated mutations on glucokinase activity and protein stability. Biochem. J..

[13] Garcia-Herrero C.M., Galan M., Vincent O., Flandez B., Gargallo M., Delgado-Alvarez E., Blazquez E., Navas M.A. (2007). Functional analysis of human glucokinase gene mutations causing MODY2: exploring the regulatory mechanisms of glucokinase activity. Diabetologia.

[14] Gloyn A.L., Odili S., Zelent D., Buettger C., Castleden H.A., Steele A.M., Stride A., Shiota C., Magnuson M.A., Lorini R. (2005). Insights into the structure and regulation of glucokinase from a novel mutation (V62M), which causes maturity-onset diabetes of the young. J. Biol. Chem..

[15] Sagen J.V., Odili S., Bjorkhaug L., Zelent D., Buettger C., Kwagh J., Stanley C., Dahl-Jorgensen K., de Beaufort C., Bell G.I. (2006). From clinicogenetic studies of maturity-onset diabetes of the young to unraveling complex mechanisms of glucokinase regulation. Diabetes.

[16] Davis E.A., Cuesta-Munoz A., Raoul M., Buettger C., Sweet I., Moates M., Magnuson M.A., Matschinsky F.M. (1999). Mutants of glucokinase cause hypoglycaemia- and hyperglycaemia syndromes and their analysis illuminates fundamental quantitative concepts of glucose homeostasis. Diabetologia.

[17] Liang Y., Kesavan P., Wang L.Q., Niswender K., Tanizawa Y., Permutt M.A., Magnuson M.A., Matschinsky F.M. (1995). Variable effects of maturity-onset-diabetes-of-youth (MODY)-associated glucokinase mutations on substrate interactions and stability of the enzyme. Biochem. J..

[18] Kesavan P., Wang L., Davis E., Cuesta A., Sweet I., Niswender K., Magnuson M.A., Matschinsky F.M. (1997). Structural instability of mutant beta-cell glucokinase: implications for the molecular pathogenesis of maturity-onset diabetes of the young (type-2). Biochem. J..

[19] Miller S.P., Anand G.R., Karschnia E.J., Bell G.I., LaPorte D.C., Lange A.J. (1999). Characterization of glucokinase mutations associated with maturity-onset diabetes of the young type 2 (MODY-2): different glucokinase defects lead to a common phenotype. Diabetes.

[20] Pino M.F., Kim K.A., Shelton K.D., Lindner J., Odili S., Li C., Collins H.W., Shiota M., Matschinsky F.M., Magnuson M.A. (2007). Glucokinase thermolability and hepatic regulatory protein binding are essential factors for predicting the blood glucose phenotype of missense mutations. J. Biol. Chem..

[21] Liang Y., Najafi H., Smith R.M., Zimmerman E.C., Magnuson M.A., Tal M., Matschinsky F.M. (1992). Concordant glucose induction of glucokinase, glucose usage, and glucose-stimulated insulin release in pancreatic islets maintained in organ culture. Diabetes.

[22] Flanagan S.E., De Franco E., Lango Allen H., Zerah M., Abdul-Rasoul M.M., Edge J.A., Stewart H., Alamiri E., Hussain K., Wallis S. (2014). Analysis of transcription factors key for mouse pancreatic development establishes NKX2–2 and MNX1 mutations as causes of neonatal diabetes in man. Cell Metab..

[23] Ng P.C., Henikoff S. (2001). Predicting deleterious amino acid substitutions. Genome Res..

[24] Ramensky V., Bork P., Sunyaev S. (2002). Human non-synonymous SNPs: server and survey. Nucleic Acids Res..

[25] Gonzalez-Perez A., Lopez-Bigas N. (2011). Improving the assessment of the outcome of nonsynonymous SNVs with a consensus deleteriousness score, Condel. Am. J. Hum. Gen..

[26] Kamata K., Mitsuya M., Nishimura T., Eiki J., Nagata Y. (2004). Structural basis for allosteric regulation of the monomeric allosteric enzyme human glucokinase. Structure.

[27] Greenbaum A.L., Gumaa K.A., McLean P. (1971). The distribution of hepatic metabolites and the control of the pathways of carbohydrate metabolism in animals of different dietary and hormonal status. Arch. Biochem. Biophys..

[28] Ellard S., Lango Allen H., De Franco E., Flanagan S.E., Hysenaj G., Colclough K., Houghton J.A., Shepherd M., Hattersley A.T., Weedon M.N. (2013). Improved genetic testing for monogenic diabetes using targeted next-generation sequencing. Diabetologia.

[29] Beer N.L., Osbak K.K., van de Bunt M., Tribble N.D., Steele A.M., Wensley K.J., Edghill E.L., Colcough K., Barrett A., Valentinova L. (2012). Insights into the pathogenicity of rare missense GCK variants from the identification and functional characterization of compound heterozygous and double mutations inherited in cis. Diabetes Care.

[30] Steele A.M., Tribble N.D., Caswell R., Wensley K.J., Hattersley A.T., Gloyn A.L., Ellard S. (2011). The previously reported T342P GCK missense variant is not a pathogenic mutation causing MODY. Diabetologia.

[31] Shen Y., Cai M., Liang H., Wang H., Weng J. (2011). Insight into the biochemical characteristics of a novel glucokinase gene mutation. Hum. Genet..

[32] Fenner D., Odili S., Hong H.K., Kobayashi Y., Kohsaka A., Siepka S.M., Vitaterna M.H., Chen P., Zelent B., Grimsby J. (2011). Generation of N-ethyl-N-nitrosourea (ENU) diabetes models in mice demonstrates genotype-specific action of glucokinase activators. J. Biol. Chem..

[33] Beer N.L., Tribble N.D., McCulloch L.J., Roos C., Johnson P.R., Orho-Melander M., Gloyn A.L. (2009). The P446L variant in GCKR associated with fasting plasma glucose and triglyceride levels exerts its effect through increased glucokinase activity in liver. Hum. Mol. Genet..

[34] Christesen H.B., Jacobsen B.B., Odili S., Buettger C., Cuesta-Munoz A., Hansen T., Brusgaard K., Massa O., Magnuson M.A., Shiota C. (2002). The second activating glucokinase mutation (A456V): implications for glucose homeostasis and diabetes therapy. Diabetes.

