# Supplementary material for: Phenotypic severity of homozygous GCK mutations causing neonatal or childhood-onset diabetes is primarily mediated through effects on protein stability
Source: Hum Mol Genet. 2014 Jul 11;23(24):6432–40. doi: 10.1093/hmg/ddu360 (PMC4240195; doi:10.1093/hmg/ddu360)
Supplement: Supplementary Data [file supp_23_24_6432__index.html]

Phenotypic severity of homozygous GCK mutations causing neonatal or childhood-onset diabetes is primarily mediated through effects on protein stability — Phenotypic severity of homozygous GCK mutations causing neonatal or childhood-onset diabetes is primarily mediated through effects on protein stability — Supplementary Data 

# Phenotypic severity of homozygous *GCK* mutations causing neonatal or childhood-onset diabetes is primarily mediated through effects on protein stability

## Supplementary Data

Supplementary Data

**Files in this Data Supplement:**

- Supplementary Data - Docx file
- Supplementary Table 1 - docx file
- Supplementary Table 2 - docx file
- Supplementary Table 3 - docx file
