# Supplementary material for: Phenotypic severity of homozygous GCK mutations causing neonatal or childhood-onset diabetes is primarily mediated through effects on protein stability
Source: Hum Mol Genet. 2014 Jul 11;23(24):6432–40. doi: 10.1093/hmg/ddu360 (PMC4240195; doi:10.1093/hmg/ddu360)

**Supplementary Figure 1.** Pedigrees for the two childhood-onset GCK mutations. Squares and circles denote males and females respectively. Filled symbols represent diabetic individuals, grey symbols MODY individuals, open symbols healthy individuals, and hatched symbols individuals with a history of gestational diabetes. An arrow indicates the proband in each family. Where no genetic result is given, testing was not performed. N, no mutation; NT, not tested; DM, diabetes mellitus; BMI, body mass index; N/A, data not available.

**Supplementary Figure 2.** Linear regression analysis of the relationship between age-at-diagnosis and birth weight standard deviation score (Birth Weight SDS) for homozygous *GCK* mutation carriers.


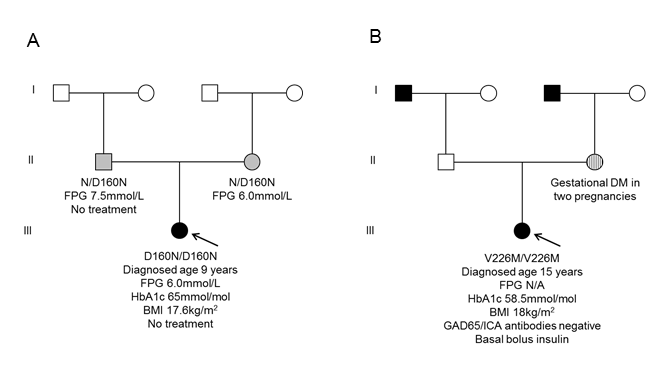


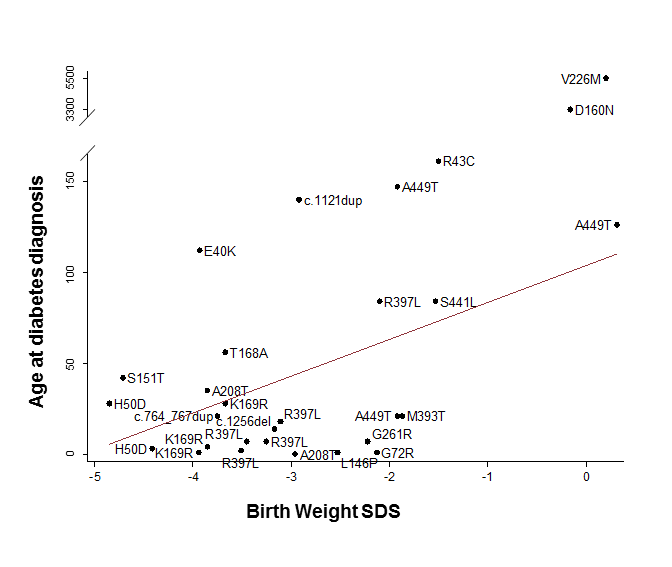

Supplement: Supplementary Data [file supp_ddu360_ddu360supp.docx]
