# Supplementary material for: Phenotypic severity of homozygous GCK mutations causing neonatal or childhood-onset diabetes is primarily mediated through effects on protein stability
Source: Hum Mol Genet. 2014 Jul 11;23(24):6432–40. doi: 10.1093/hmg/ddu360 (PMC4240195; doi:10.1093/hmg/ddu360)
Supplement: Supplementary Data [file supp_ddu360_ddu360supp_table1.docx]

| **Protein** | **Functional assessment (RAI)** | **Thermostability assessment** | **PolyPhen-2** | **SIFT** | **Condel** |
| --- | --- | --- | --- | --- | --- |
| **E40K** | Damaging | Decreased | Probably damaging | Damaging | Deleterious |
| **R43C** | Damaging | Decreased | Probably damaging | Damaging | Deleterious |
| **H50D** | Damaging | Decreased | Possibly damaging | Damaging | Neutral |
| **G72R** | Neutral | Decreased | Probably damaging | Damaging | Deleterious |
| **L146P** | Damaging | N/A | Probably damaging | Damaging | Deleterious |
| **G68D** | Neutral | WT-like | Probably damaging | Damaging | Deleterious |
| **S151T** | Damaging | N/A | Probably damaging | Damaging | Deleterious |
| **D160N** | Damaging | Increased^§^ | Probably damaging | Damaging | Neutral |
| **T168A** | Damaging | N/A | Probably damaging | Damaging | Deleterious |
| **K169R** | Damaging | N/A | Probably damaging | Damaging | Deleterious |
| **A208T** | Damaging | N/A | Probably damaging | Damaging | Deleterious |
| **V226M** | Damaging | Increased^§^ | Probably damaging | Damaging | Deleterious |
| **G261R** | Damaging | N/A | Probably damaging | Damaging | Deleterious |
| **T342P** | Neutral | WT-like | Benign | Tolerated | Neutral |
| **M393T** | Neutral | Decreased | Possibly damaging | Damaging | Deleterious |
| **R397L** | Neutral | Decreased | Probably damaging | Damaging | Deleterious |
| **S441L** | Damaging | Decreased | Probably damaging | Damaging | Deleterious |
| **A449T** | Activating | Decreased | Probably damaging | Damaging | Deleterious |

**Supplementary Table 1.** Comparison of bioinformatic predictions with functional effects for human mutant GST-GCK proteins. Variants were assessed using the PolyPhen v.2.2.2, SIFT Human Protein, and Condel web server algorithms. N/A, enzyme not analyzed for thermostability effects due to negligible activity in kinetic assays. ^§^as indicated by the improved stability profile for this protein in thermostability assays.
