# Supplementary material for: Phenotypic severity of homozygous GCK mutations causing neonatal or childhood-onset diabetes is primarily mediated through effects on protein stability
Source: Hum Mol Genet. 2014 Jul 11;23(24):6432–40. doi: 10.1093/hmg/ddu360 (PMC4240195; doi:10.1093/hmg/ddu360)
Supplement: Supplementary Data [file supp_ddu360_ddu360supp_table2.docx]

| **Mutation** | **BW SDS score** | **Age-at-diagnosis score** | **Clinical Severity Score** | **Clinical Severity Grade** |
| --- | --- | --- | --- | --- |
| **D160N** | 1 | 1 | 2 | Very Mild |
| **V226M** | 1 | 1 | 2 | Very Mild |
| **R43C** | 1 | 2 | 3 | Mild |
| **S441L** | 1 | 2 | 3 | Mild |
| **A449T** | 1 | 2.3 | 3.3 | Mild |
| **c.1121dup** | 2 | 2 | 4 | Mild |
| **M393T** | 1 | 3 | 4 | Mild |
| **T168A** | 3 | 1.5 | 4.5 | Moderate |
| **G261R** | 2 | 4 | 6 | Moderate |
| **E40K** | 4 | 2 | 6 | Moderate |
| **G72R** | 2 | 4 | 6 | Moderate |
| **L146P** | 2 | 4 | 6 | Moderate |
| **S151T** | 4 | 2 | 6 | Moderate |
| **A208T** | 3 | 3 | 6 | Moderate |
| **R397L** | 2.6 | 3.4 | 6 | Moderate |
| **c.764_767dup** | 3 | 3 | 6 | Moderate |
| **c.1256del** | 3 | 3 | 6 | Moderate |
| **H50D** | 4 | 3.5 | 7.5 | Severe |
| **K169R** | 4 | 3.7 | 7.6 | Severe |

**Supplementary Table 2.** Clinical severity score and grade by mutation. Clinical Severity Grades were assigned according to Clinical Severity Scores (CSSs) as follows: Very Mild CSS<2; Mild 2<CSS≤4; Moderate 4<CSS≤6; Severe CSS>6. Where there is data for more than one patient, the score is based on the average BW SDS and age-at-diagnosis values for that mutation.
