# Supplementary material for: Phenotypic severity of homozygous GCK mutations causing neonatal or childhood-onset diabetes is primarily mediated through effects on protein stability
Source: Hum Mol Genet. 2014 Jul 11;23(24):6432–40. doi: 10.1093/hmg/ddu360 (PMC4240195; doi:10.1093/hmg/ddu360)
Supplement: Supplementary Data [file supp_ddu360_ddu360supp_table3.docx]

| **Protein** | **Yield (mg)** | **S0.5 (mmol/L)** | **Hill (nH)** | **ATPKm (mmol/L)** | **Kcat a (s-1)** | **Kcat b (s-1)** | **RAI** | **RSI** | **TA50 (°C)** |
| --- | --- | --- | --- | --- | --- | --- | --- | --- | --- |
| **WT** | 31.25 | 7.98 ± 0.16 | 1.58 ± 0.02 | 0.51 ± 0.01 | 61.47 ± 0.32 | 49.50 ± 0.56 | 1.00 | 1.00 | 54.89 |
| **E40K^¶^** | 3.28 | 20.91 ± 0.23 | 1.61 ± 0.01 | 0.51 ± 0.01 | 19.98 ± 0.42 | 13.99 ± 0.12 | 0.02 | 0.11 | 49.94 |
| **R43C** | 3.37 | 8.77 ± 0.10 | 1.70 ± 0.01 | 0.48 ± 0.01 | 27.24 ± 0.19 | 27.49 ± 0.33 | 0.26 | 0.37 | 51.37 |
| **H50D^¶^** | 3.88 | 19.40 ± 0.23 | 1.74 ± 0.02 | 0.52 ± 0.02 | 22.79 ± 0.22 | 22.87 ± 0.14 | 0.02 | 0.00 | 49.30 |
| **G72R**^\|^ | 6.58 | 7.39 ± 0.09 | 1.34 ± 0.01 | 0.91 ± 0.02 | 34.40 ± 0.33 | 37.17 ± 0.30 | 1.05 | 0.34 | 51.24 |
| **G68D** | 15.33 | 5.97 ± 0.06 | 1.75 ± 0.05 | 0.37 ± 0.01 | 38.38 ± 0.24 | 44.59 ± 0.71 | 1.00 | 0.94 | 54.54 |
| **L146P** | 5.24 | N/A | N/A | N/A | N/A | N/A | N/A | N/A | N/A |
| **S151T** | 20.38 | N/A | N/A | N/A | N/A | N/A | N/A | N/A | N/A |
| **D160N** | 16.15 | 12.44 ± 0.12 | 1.71 ± 0.01 | 0.27 ± 0.01 | 26.40 ± 0.25 | 29.70 ± 0.36 | 0.09 | 0.98^**^ | 54.75 |
| **T168A^¶,^**^\|^ | 12.88 | 244.60 ± 8.25 | 0.77 ± 0.01 | 8.01 ± 0.18 | 6.75 ± 0.09 | 12.56 ± 0.14 | <0.01 | N/A | N/A |
| **K169R** | 2.95 | N/A | N/A | N/A | N/A | N/A | N/A | N/A | N/A |
| **A208T^¶,^**^\|^ | 35.17 | 81.76 ± 2.77 | 0.87 ± 0.01 | 2.76 ± 0.05 | 9.05 ± 0.11 | 14.28 ± 0.11 | 0.01 | N/A | N/A |
| **V226M^¶,^**^\|^ | 8.96 | 43.24 ± 0.44 | 1.08 ± 0.004 | 2.37 ± 0.04 | 53.80 ± 0.52 | 88.43 ± 1.30 | 0.07 | 0.81^**^ | 53.82 |
| **G261R^¶^** | 0.52 | 281.83 ± 6.14 | 1.50 ± 0.02 | 0.64 ± 0.01 | 24.39 ± 0.30 | 19.99 ± 0.25 | <0.01 | N/A | N/A |
| **T342P** | 16.77 | 8.37 ± 0.11 | 1.69 ± 0.01 | 0.52 ± 0.02 | 71.11 ± 0.40 | 60.97 ± 0.55 | 0.77 | 1.01 | 54.93 |
| **M393T** | 12.37 | 6.41 ± 0.06 | 1.69 ± 0.01 | 0.50 ± 0.01 | 65.19 ± 0.38 | 57.73 ± 0.30 | 1.52 | 0.12 | 49.98 |
| **R397L** | 16.18 | 6.67 ± 0.06 | 1.68 ± 0.01 | 0.50 ± 0.01 | 64.38 ± 0.48 | 58.27 ± 0.40 | 1.38 | 0.08 | 49.75 |
| **S441L** | 27.96 | 8.55 ± 0.13 | 1.59 ± 0.01 | 0.71 ± 0.01 | 40.95 ± 0.22 | 33.44 ± 0.20 | 0.51 | 0.37 | 51.38 |
| **A449T**^\|^ | 15.23 | 2.03 ± 0.03 | 1.38 ± 0.02 | 1.76 ± 0.03 | 8.47 ± 0.04 | 11.37 ± 0.11 | 2.36 | 0.43 | 51.68 |

**Supplementary Table 3.** Kinetic characterization of mutant GCK enzymes. Glucose affinity (S0.5), Hill number (nH), and turnover number (Kcat) values were determined in the presence of 0-100mmol/L glucose (**^¶^**except for indicated variants as described in Materials and Methods). Affinity for ATP (ATPKm) was determined in the presence of 0-5mmol/L ATP (^|^except for indicated variants as described in Materials and Methods). Kcat a values were determined from glucose S0.5 assays, and Kcat b values from ATPKm assays. Relative activity indices (RAI), Relative Stability Indices (RSI) and TA50 values were calculated as described in Materials and Methods. Data shown are mean ±SEM (n=3). N/A, data unobtainable as mutation resulted in a protein with such poor affinity for glucose and/or ATP that its functional and/or thermostability characteristics could not be determined; ^**^RSI value unable to capture the improved stability profile for this protein at temperatures <TA50.
